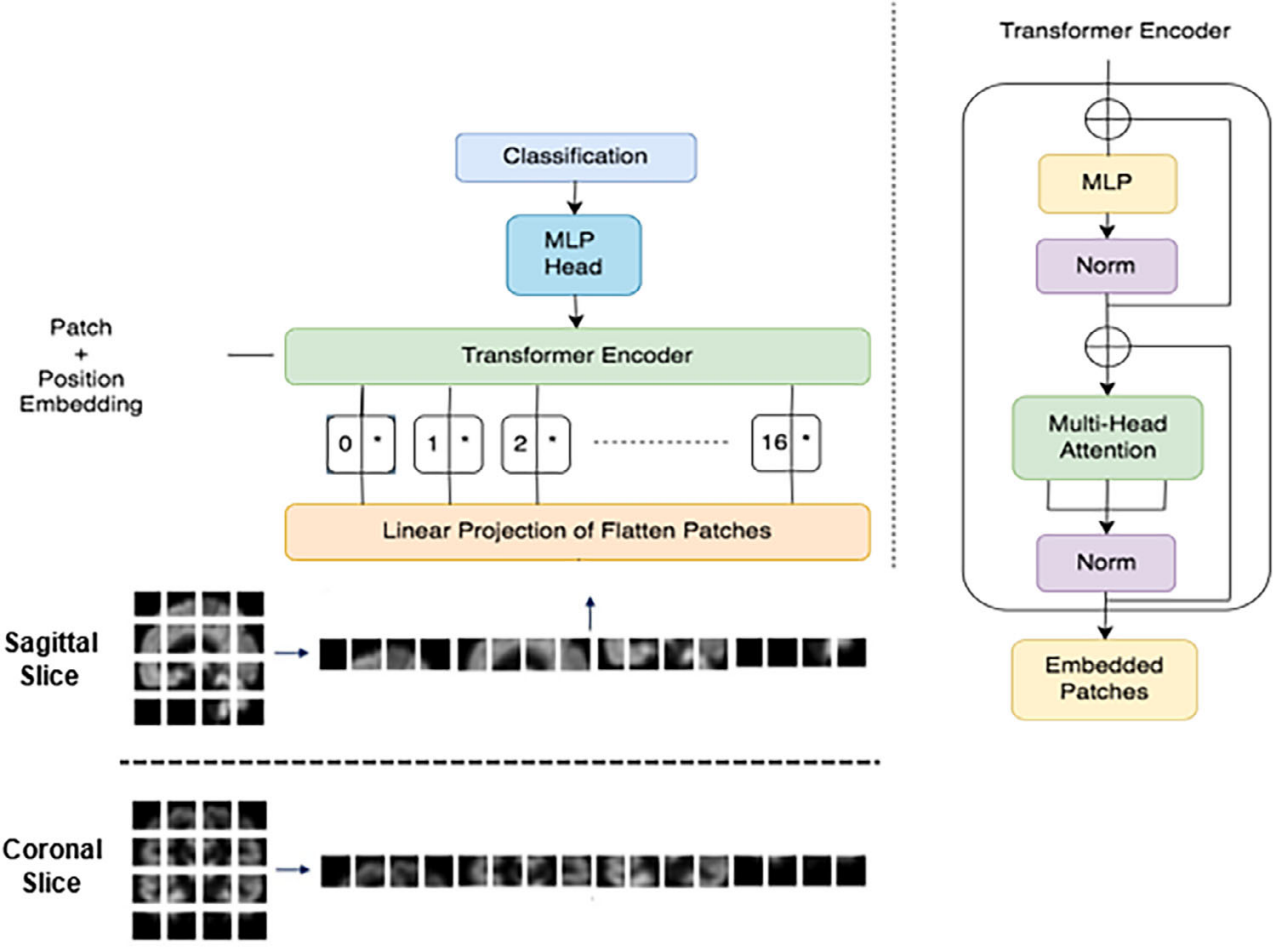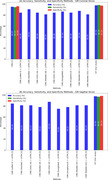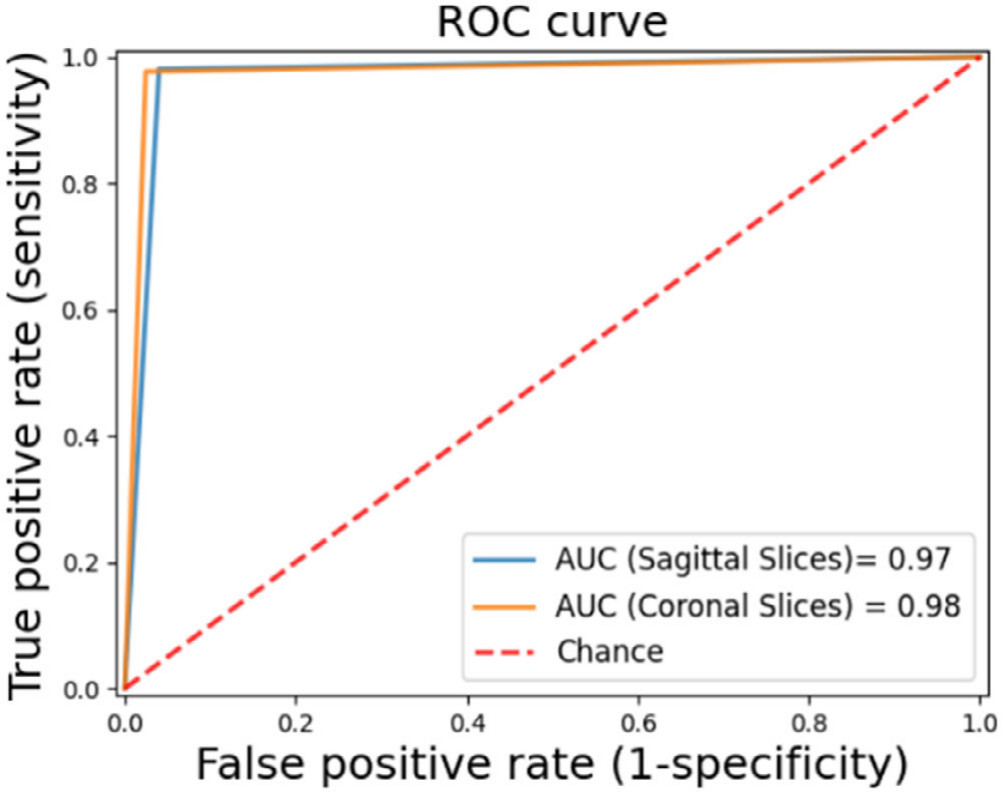# Alzheimer’s disease diagnosis using gray matter of T1‐weighted sMRI data and vision transformer

**DOI:** 10.1002/alz.089944

**Published:** 2025-01-09

**Authors:** Maryam Akhavan Aghdam, Serdar Bozdag, Fahad Saeed

**Affiliations:** ^1^ Florida International University, Miami, FL USA; ^2^ BioDiscovery Institute / University of North Texas, Denton, TX USA; ^3^ University of North Texas, Denton, TX USA

## Abstract

**Background:**

Alzheimer's Disease (AD) is a progressive neurodegenerative disorder characterized by memory loss and cognitive decline. Traditional diagnostic methods, mainly based on cognitive, memory, and behavioral tests, have limitations, particularly in the early detection of AD. Structural magnetic resonance imaging (sMRI) has emerged as a key tool in understanding the brain changes associated with AD, focusing particularly on alterations in gray matter (GM). However, the complexity of brain changes in AD requires sophisticated analysis methods. In recent years, machine learning (ML) models have shown great potential in interpreting complex neuroimaging data. These models can detect intricate patterns in neuroimaging data, making them invaluable in enhancing the diagnostic accuracy and early AD diagnosis. Therefore, combining the neuroimaging data with ML models presents a promising direction in improving the early‐diagnosis and understanding of AD.

**Method:**

We propose a novel approach to diagnose AD using Vision Transformer (ViT) (Figure 1) [1], a cutting‐edge class of ML model, and GM of T1‐weighted sMRI data. The proposed approach leverages the power of deep‐learning (DL) to detect the GM changes that are indicative of AD. We used pretrained ViT model to extract features from the GM sagittal and coronal slices of sMRI data and classify AD from cognitively normal (CN). We employed ADNI dataset, focusing on subjects with T1‐weighed MPRAGE sMRI scans, including 70 AD patients and 85 CN individuals.

**Result:**

The study achieved an average classification accuracy of 97.6% in sagittal slices and 97.7% in coronal slices (Figures 2 and 3). These results indicate a significantly higher accuracy in diagnosing AD using the proposed method compared to other state‐of‐the‐art models based on sMRI data. The high accuracy underscores the model's capability in effectively distinguishing between AD patients and CN individuals, demonstrating its potential utility in clinical settings.

**Conclusion:**

The proposed approach demonstrates a significant advancement in the accurate diagnosis of AD, which might be useful for early‐diagnosis. Our proposed ML model represents a considerable improvement over existing ML methods, offering a new avenue for research and application in the field of neurodegenerative diseases.